# Reproductive hormones, bone mineral content, body composition, and testosterone therapy in boys and adolescents with Klinefelter syndrome

**DOI:** 10.1530/EC-23-0031

**Published:** 2023-06-12

**Authors:** Hans Valdemar López Krabbe, Jørgen Holm Petersen, Louise Laub Asserhøj, Trine Holm Johannsen, Peter Christiansen, Rikke Beck Jensen, Line Hartvig Cleemann, Casper P Hagen, Lærke Priskorn, Niels Jørgensen, Katharina M Main, Anders Juul, Lise Aksglaede

**Affiliations:** 1 Department of Growth and Reproduction, Copenhagen University Hospital, Rigshospitalet, Copenhagen, Denmark; 2 International Centre for Research and Research Training in Endocrine Disruption of Male Reproduction and Child Health (EDMaRC), Copenhagen University Hospital - Rigshospitalet, Copenhagen, Denmark; 3 Section of Biostatistics, University of Copenhagen, Copenhagen, Denmark; 4 Department of Fertility, Copenhagen University Hospital, Rigshospitalet, Copenhagen, Denmark; 5 Department of Clinical Medicine, University of Copenhagen, Copenhagen, Denmark

**Keywords:** Klinefelter syndrome, body composition, truncal obesity, bone mineral content

## Abstract

Adult patients with Klinefelter syndrome (KS) are characterized by a highly variable phenotype, including tall stature, obesity, and hypergonadotropic hypogonadism, as well as an increased risk of developing insulin resistance, metabolic syndrome, and osteoporosis. Most adults need testosterone replacement therapy (TRT), whereas the use of TRT during puberty has been debated. In this retrospective, observational study, reproductive hormones and whole-body dual-energy x-ray absorptiometry-derived body composition and bone mineral content were standardized to age-related standard deviation scores in 62 patients with KS aged 5.9–20.6 years. Serum concentrations of total testosterone and inhibin B were low, whereas luteinizing hormone and follicle-stimulating hormone were high in patients before TRT. Despite normal body mass index, body fat percentage and the ratio between android fat percentage and gynoid fat percentage were significantly higher in the entire group irrespective of treatment status. In patients evaluated before and during TRT, a tendency toward a more beneficial body composition with a significant reduction in the ratio between android fat percentage and gynoid fat percentage during TRT was found. Bone mineral content (BMC) did not differ from the reference, but BMC corrected for bone area was significantly lower when compared to the reference. This study confirms that patients with KS have an unfavorable body composition and an impaired bone mineral status already during childhood and adolescence. Systematic studies are needed to evaluate whether TRT during puberty will improve these parameters.

## Introduction

Klinefelter syndrome (KS) is the most frequent sex chromosome aneuploidy in men, showing a karyotype of 47,XXY and having an inconsistent adult phenotype comprising tall stature, obesity, and hypergonadotropic hypogonadism, as well as an increased risk of developing insulin resistance, metabolic syndrome, and osteoporosis (1). Low to low-normal serum concentrations of testosterone are seen in most adults with KS, but nearly all have highly elevated concentrations of luteinizing hormone (LH) and follicle-stimulating hormone (FSH) combined with unmeasurable inhibin B, which reflects their primary testicular defect (2, 3, 4). Reduced testosterone concentrations may contribute to the well-known reduction of bone mineral density (BMD), increase of body fat mass, and decrease of muscle and lean mass with subsequent higher risk of developing osteoporosis and type 2 diabetes. However, the underlying mechanism behind these phenotypic traits in KS remains unclear. Recent studies have identified genome-wide alterations affecting both autosomes and sex chromosomes, indicating that a complex combination of the intrinsic genetic background, hypogonadism, as well as life style factors is involved and may potentially explain some of the variability in the KS phenotype (1, 5, 6, 7).

Studies on body composition in children with KS have shown that already during early infancy and before puberty, these boys may exhibit an unfavorable body composition (8, 9, 10, 11). Furthermore, impaired bone mineralization has been reported in some (12, 13) but not all studies of children with KS (11). Early prevention is important, and detailed description and follow-up on boys with KS is important to better understand the natural history and thereby to develop guidelines for optimal treatment of this patient group. We therefore aimed to describe reproductive hormones, anthropometry, and dual-energy x-ray absorptiometry (DXA)-derived body composition and bone mineral content (BMC) in 62 patients with KS aged 5.9–20.6 years.

## Materials and methods

### Study population

#### Patients with Klinefelter syndrome

Patients eligible for this retrospective, observational tertiary center study at The Department of Growth and Reproduction, Rigshospitalet, Copenhagen, Denmark, had non-mosaic 47,XXY KS and had undergone whole-body DXA and/or had minimum one serum concentration of testosterone determined by liquid chromatography-tandem mass spectrometry (LC-MS/MS) methodology. Patients suffering from other conditions potentially affecting growth and development were excluded. A total of 65 patients met the inclusion criteria, but 3 patients were excluded due to trisomy 21 (*n* = 1), previous chemotherapy (*n* = 1), and severe skeletal disorder (*n* = 1), leaving a total of 62 patients aged 5.9–20.6 years for analysis.

In total, 21 patients were diagnosed prenatally, and 41 patients were diagnosed postnatally due to psychosocial or cognitive issues (*n* = 21), small testis volume/reduced virilization (*n* = 9), gynecomastia (*n* = 5), early puberty (*n* = 1), tall stature (*n* = 1), or hypotelorism (*n* = 1). The reason for diagnosis was unknown in three patients. Median age at postnatal diagnosis was 14.5 years (1–19 years). The patients were examined from August 2008 to June 2022.

#### Controls

A total of 2823 healthy boys and adolescents aged 7.7–20.0 (median 18.8) years were recruited from two population-based studies and served as controls for the development of Danish reference curves for body composition and bone mineralization based on data derived from whole-body DXA. Boys (*n* = 535) aged 7.7–14.3 (median 10.8) years were part of the Copenhagen Mother–Child Cohort, a population-based longitudinal birth cohort of healthy Danish children (14). Data on body fat percentage in these boys has previously been published using a standard algorithm (15). Adolescent males (*n* = 2288) aged 18.0–20.0 (median 18.9) years were recruited at the compulsory physical examination for military service as part of a cross-sectional study on young men from the general population (16).

### Anthropometry

Standing height was measured to the nearest 0.1 cm using a wall-mounted stadiometer, and weight was measured on a digital electronic scale with a precision of 0.1 kg. Body mass index (BMI, weight/height^2^ (kg/m^2^)) was calculated. The patients were measured and weighed on the same day as DXA was performed.

### Whole-body DXA

Whole-body DXA scans were performed using Lunar Prodigy (GE Healthcare) and analyzed using enCORE software enhanced, version 16. For quality control, calibration was performed as recommended by the manufacturer using a quality assurance block daily and using a spine phantom on a weekly basis.

A total of 88 whole-body DXA scans were available in 51 patients with KS, resulting in an average of 1.7 (range 1–4) scans per patient. In patients older than 20.0 years (*n* = 3), data on total body less head (TBLH) values were not available. In some patients, DXA was performed only before (*n* = 15) or only during (*n* = 19) TRT, whereas others were evaluated before and during TRT (*n* = 17).

DXA-derived data regarding bone mineralization included TBLH BMC (BMC for age), TBLH bone mineral density (BMD) (BMD for age), TBLH BMC adjusted for TBLH bone area (BMC for bone area), and TBLH bone area adjusted for height (bone area for height). Body composition was expressed as lean body mass (defined as body weight minus body fat mass minus bone mass), body fat percentage (defined as body fat mass/(body weight minus bone mass)), android fat percentage (visceral fat percentage), gynoid fat percentage (fat percentage on hips and thighs), and ratio between android fat percentage and gynoid fat percentage.

### Hormone analyses

Serum concentrations of total testosterone, total estradiol (total E2), sex hormone-binding globulin (SHBG), FSH, LH, anti-Müllerian hormone (AMH), and inhibin B were measured. All analyses were accredited by The Danish Accreditation Fund for medical examination according to a European and international standard approved in Denmark (the standard DS/EN ISO 15189).

Blood samples were available from a total of 47 patients with KS with an average number of 4.5 samples (range 1–13) per patient. The serum concentrations of total testosterone and LH were available in all patients, whereas FSH, inhibin B, AMH, total E2, and free E2 were available in 42, 42, 38, 31, and 31, respectively. Most patients (*n* = 37) had blood samples taken before starting TRT, some were already on TRT at the first evaluation (*n* = 10), and others started on TRT during follow-up (*n* = 21).

Total testosterone and total E2 were measured by LC-MS/MS. The limit of quantification (LOQ) and inter-assay coefficient of variation (CV) for total testosterone were 0.10 nmol/L and <2.5%, respectively (17). LOQ and CV for total E2 were 12 pmol/L and <7% , respectively (18). Free testosterone was calculated using the formula by Vermeulen (19), whereas free E2 was calculated based on Mazer (20). Until 2014, serum SHBG was measured by a time-resolved immunofluorescence assay (AutoDelfia; Wallac Oy, Turku, Finland) with a limit of detection (LOD) and CV of 0.23 nmol/L and <6%, while SHBG from 2014 and onward was measured by a chemiluminescence immunoassay (Access2, Beckman Coulter) (all patients with KS) with an LOD of 0.35 nmol/L and CV <5%. All AutoDELFIA-derived SHBG results were factored to corresponding Access2-derived SHBG results after internal method comparison.

Until 2022, FSH and LH were analyzed by time-resolved immunofluorometric assays (AutoDelfia; PerkinElmer), both with LODs of 0.05 IU/L and CVs below 9%, as previously described (21). From 2022 and onward, FSH and LH were analyzed by chemiluminescence immunoassays (Atellica; Siemens Healthineers) with LODs of LH of 0.07 IU/L and FSH of 0.3 U/L, respectively, and CVs <25% at low concentrations. FSH results from the immunofluorometric method were recalculated to the equivalent results from the chemiluminometric method using an internal correction factor to adjust for instrument bias, while no correction was required between the LH methods.

AMH was measured by AMH-enzyme-linked immunosorbent assay (ELISA) (Immunotech, Beckman Coulter) until 2015 and by chemiluminescence immunoassay (Access2, Beckman Coulter) from 2015 and onward. Based on an internal validation, the results of the two methods were comparable. The LOD and CV for AMH were 0.14 pmol/L and <2.5%, respectively.

An ELISA was used to determine inhibin B (Beckman Coulter Inhibin B Gen II ELISA, Beckman Coulter). The LOD and CV for inhibin B was 3 pg/mL and 11%, respectively.

Reproductive hormone measurements below LODs were replaced with ½LOD for graphic illustration and for calculation of SDS, free testosterone, and free E2.

### Clinical follow-up and TRT

The care of patients with KS is a highly specialized function, which is handled in the three tertiary centers of pediatric endocrinology in Denmark. Boys with KS are followed with annual or biennial visits during childhood. The clinical examination is individualized but generally includes anthropometric measurements, testicular evaluation, rough neurological evaluation, and discussion about the need for further psychological evaluation. At the time of predicted puberty, the frequency of visits increases on an individualized basis, and the clinical examination at this age includes assessment of pubertal development, anthropometric measurements, measurement of reproductive hormones, and evaluation of symptoms of testosterone deficiency (e.g. gynecomastia, fatigue, low muscle tone, and obesity). Whole-body DEXA scan is performed every second to third year during puberty but may be performed at an earlier age on an individualized basis (e.g. due to limited intake of calcium and vitamin D and concerns about bone health or in boys with a low BMI but signs of a high fat percentage).

In our department, it is gold standard to start TRT in boys with KS around the time of puberty. TRT is initiated after clinical onset of puberty, as assessed according to the methods by Marshall & Tanner (22) either if the boy exhibits symptoms of testosterone deficiency or if the serum concentration of LH exceeds +2 s.d.. Standard treatment is either peroral testosterone undecanoate (TU) (Testosterone ‘Paranova’; (Paranova Danmark A/S, Herlev, Denmark)) or transdermal testosterone (Tostran 2%; Kyowa Kirin, Holland), with dose titration according to serum concentrations of testosterone and a target of a serum testosterone in the upper half of the reference range. The starting dose of peroral TU is 40 mg per day, which is increased by 40 mg up to 80 mg twice daily as the maximum dose, whereas the starting dose of transdermal testosterone is either 10 mg every second day or 10 mg a day. In the beginning of the study period, peroral testosterone was frequently used, whereas transdermal testosterone has been the first choice during recent years.

### Statistics

Reference curves for DXA-derived parameters (bone mineralization and body composition) were estimated using the generalized additive model for location, scale, and shape (GAMLSS) summarizing the data in three smooth age-dependent curves: L, M, and S. The L curve adjusts for age-dependent skewness, the M curve corresponds to the age-dependent median, and the S curve is the age- dependent CV curve. The method is based on the Box–Cox power transformation, which transforms the data to follow a Gaussian distribution for each age.

To enable comparisons across ages, DXA-derived parameters, anthropometric data, as well as serum concentrations of reproductive hormones were standardized to age-related standard deviation scores (SDSs) for males using the following equation: SDS = ((*X*/*M*)^*L*^−1) / (*L* × *S*), with *X* as the measurement, *M* corresponding to the median, *L* adjusting for skewness (*L* ≠ 0), and *S* approximating the CV.

Data on bone mineralization and body composition were standardized to the new reference. SDSs of anthropometric data and serum concentrations of reproductive hormones were calculated using standard references of healthy Danish children and adolescents (17, 18, 21, 23, 24, 25). In patients more than 6 months younger than the reference material (DXA; *n* = 2, hormones; *n* = 2), SDS was not calculated. If the patient was less than 6 months younger, the youngest reference was used to calculate SDS (DXA; *n* = 1, hormones; *n* = 2).

Individual median SDSs were calculated for DXA-derived parameters, anthropometric data, and serum concentrations of reproductive hormones for each patient to avoid skewness from single patients contributing with more than one measurement. Patient SDSs were statistically compared to zero (reference material) using the one-sample *t*-test. Comparison between prenatally and postnatally diagnosed patients was performed by comparing the individual median SDSs by a two-sample *t*-test.

The treatment effect in the Klinefelter group was estimated by a multiple linear regression model, including a subject-specific effect to account for the different number of measurements for the individuals. This correctly accounts for the multiple measurements on the same individual.

Data were reported as medians and interquartile ranges. *P*-values below 0.05 were considered statistically significant. Statistical analyses were performed using IBM Statistics SPSS (version 25) and R (version 4.1.2) (https://www.R-project.org/).

### Ethics

Patients with KS and their parents (if the patient was below 18 years of age) gave informed consent for clinical and biochemical follow-up. Data from routine clinical visits were obtained from patient records and used for this study. Registration of clinical data was approved by the Danish Patient Safety Authority (no. 3-3013-1376/1/), the Team for Medical Records Research, Centre for Health, the Capital Region of Denmark (R-22031906), and the Danish Data Protection Agency (no. 2015-235 (I-Suite no. 04204) and P-2022-364).

The population-based studies were approved by the local Ethical committee of the Capital Region (RegionH) (KF 01–030/97/KF 01276357/H-1–2009–074; boys) and (journal no. H-KF-289428; adolescents) and the Danish Data Protection Agency (1997-1200-074/2005-41-5545/2010-41-4757; boys) and (2022-569; adolescents). The participants and/or their parents gave informed written consent to the study.

## Results

### Reference curves

Reference curves for DXA-derived BMC and body composition were constructed (Supplementary Fig. 1, 2 and 3, see section on supplementary materials given at the end of this article), and selected LMS values for calculating SDS are shown in Supplementary Tables 1, 2, and 3).

**Table 2:** Serum concentrations of reproductive hormones in patients with Klinefelter syndrome before and during testosterone treatment.

	Before TRT	*n*	*P*-value^a^	During TRT	*n*	*P*-value^a^
Age (years)	13.0 (11.2–15.4)	35		15.0 (13.8–16.6)	31	
Total testosterone (SDS)	–0.34 (–1.13 to 0.12)	35	*0.04*	–0.32 (–0.84 to 0.29)	31	0.12
Free testosterone (SDS)	–0.15 (–0.95 to 0.49)	35	0.15	0.42 (–0.08 to 0.81)	31	*0.02*
Total E2 (SDS)	–0.43 (–0.81 to 0.10)	22	0.26	–0.05 (–0.64 to 0.96)	20	0.48
Free E2 (SDS)	–0.02 (–0.76 to 0.37)	22	0.72	–0.02 (–0.66 to 1.25)	19	0.29
LH (SDS)	0.79 (–0.25 to 3.29)	35	*0.001*	1.87 (0.43 to 3.65)	31	*0.002*
FSH (SDS)	2.14 (0.65–3.62)	34	*<0.001*	2.98 (2.23–3.51)	27	*<0.001*
Inhibin B (SDS)	–1.35 (–4.08 to –0.24)	34	*<0.001*	–3.63 (–4.70 to –2.90)	26	*<0.001*
AMH (SDS)	0.36 (–0.98 to 1.43)	32	0.86	–0.08 (–1.54 to 0.77)	24	0.57

Data are presented as median and interquartile ranges. *P*-values in italics are significant. For boys with multiple measurements, results were averaged prior to comparison. ^a^Comparison between patients and reference.

AMH, anti-Müllerian hormone; FSH, follicle-stimulating hormone; LH, luteinizing hormone; SDS, standard deviation score; total E2, total estradiol; TRT, testosterone replacement therapy.

### Anthropometric measurements and body composition in KS

**Figure 1:**
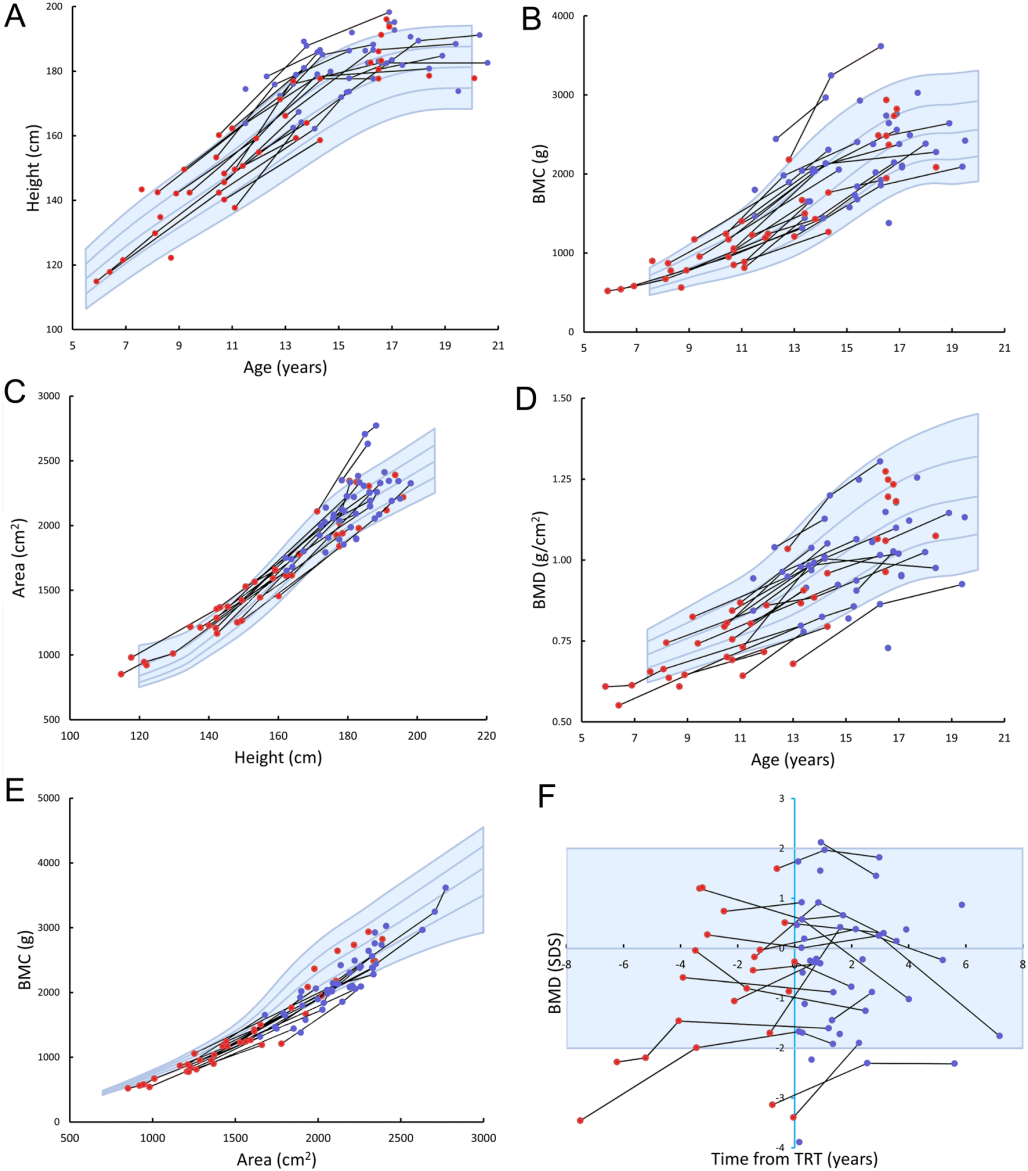
Height (A), BMC according to age (B), bone area according to height (C), BMD according to age (D), BMC according to bone area (E), and BMD SDS according to the duration of testosterone replacement therapy (F) in patients with Klinefelter syndrome. Longitudinal measures in the same individual are connected by a black line. Red dots indicate patients before testosterone replacement therapy, whereas blue dots indicate patients on testosterone replacement therapy. Blue lines and shading represent median and ±1 SDS and ±2 SDS for controls.

**Figure 2:**
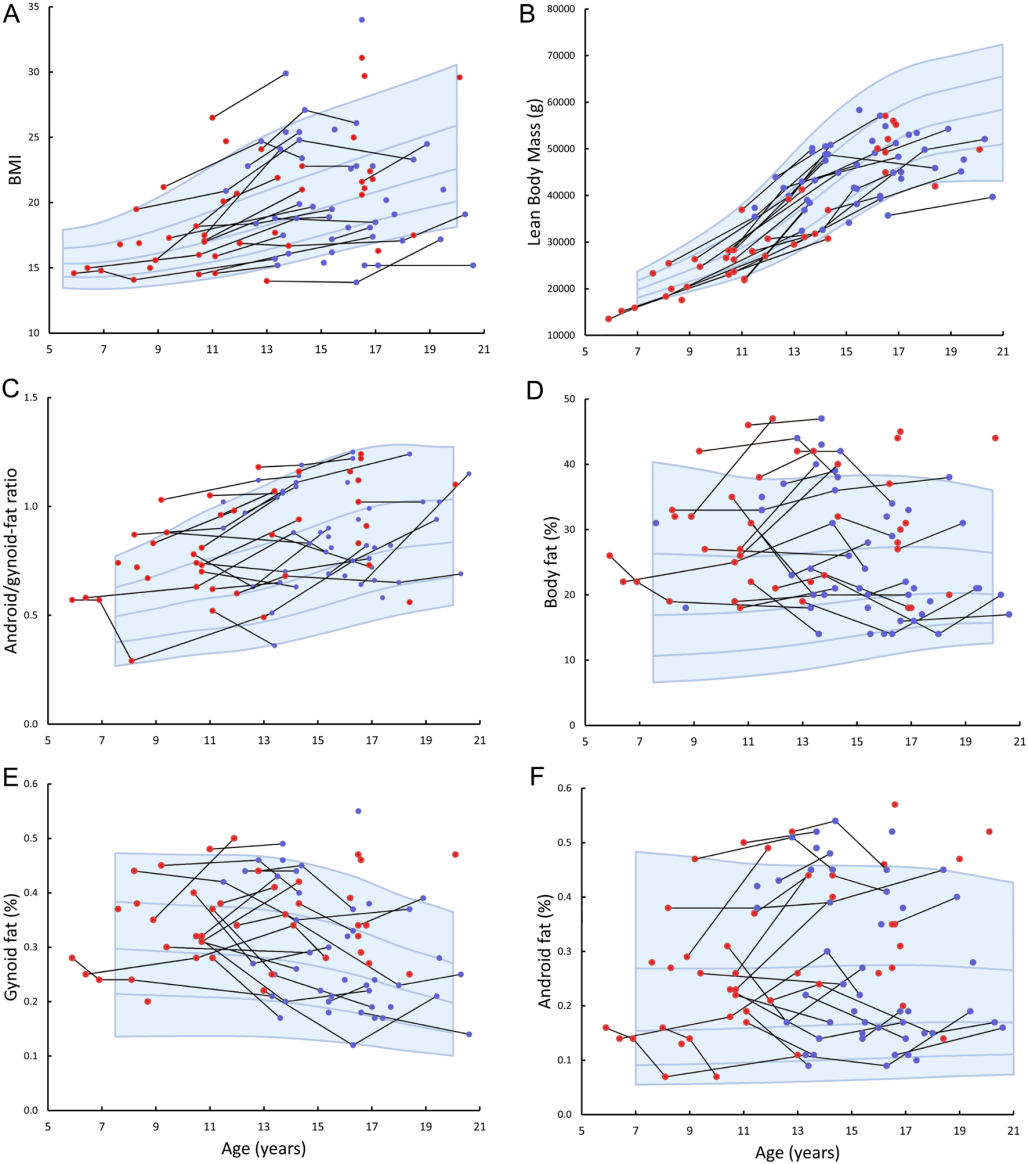
Longitudinal measures of BMI (A), lean body mass (B), ratio between android fat percentage and gynoid fat percentage (C), body fat percentage (D), gynoid fat percentage (E), and android fat percentage (F) according to age in patients with Klinefelter syndrome. Longitudinal measures in the same individual are connected by a black line. Red dots indicate patients before testosterone replacement therapy, whereas blue dots indicate patients on testosterone replacement therapy. Blue lines and shading represent median and ±1 SDS and ±2 SDS for controls.

Patients with KS were significantly taller than controls (Fig. 1A and Table 1). There were no differences in weight and BMI (Fig. 2A and Table 1). Analyzing patient data before and during TRT separately did not change the result (Table 1). There was no difference in anthropometric measures or body composition except from a significantly higher body fat percentage in prenatally diagnosed patients when comparing data from prenatally and postnatally diagnosed patients (data not shown).

**Table 1:** Anthropometry and DXA parameters in patients with Klinefelter syndrome before and during testosterone replacement treatment.

	All (*n* = 51)	*P*-value^a^	Before TRT (*n* = 32)	*P*-value^a^	During TRT (*n* = 36)	*P*-value^a^	*P*-value^b^
Age (years)	15.3 (12.4–16.6)		11.8 (9.7–16.4)		15.4 (13.8–17.1)		
Height (SDS)	0.87 (0.11–1.98)	*<0.001*	0.59 (–0.28 to 1.72)	*0.01*	1.45 (0.36–2.09)	*<0.001*	*0.002*
Weight (SDS)	0.56 (–0.74 to 1.52)	0.07	0.55 (–0.61 to 1.52)	0.05	0.19 (–0.79 to 1.65)	0.14	0.62
BMI (SDS)	0.09 (–1.08 to 1.35)	0.89	0.30 (–0.64 to 1.30)	0.27	–0.42 (–1.73 to 1.50)	0.24	0.11
Lean body mass (SDS)	–0.05 (–1.30 to 0.64)	0.07	–0.36 (–1.42 to 0.61)	*0.03*	0.09 (–1.12 to 0.89)	0.69	0.09
Body fat percentage (SDS)	1.07 (0.15–1.80)	*<0.001*	1.38 (0.52–2.01)	*<0.001*	0.28 (–0.23 to 1.74)	*0.001*	0.06
Android fat percentage (SDS)	0.97 (–0.05 to 1.76)	*<0.001*	1.04 (0.41–1.88)	*<0.001*	0.40 (–0.20 to 1.79)	*0.001*	0.22
Gynoid fat percentage (SDS)	0.81 (–0.50 to 1.58)	*<0.001*	0.94 (0.28–1.56)	*<0.001*	0.22 (–0.63 to 1.58)	0.06	0.22
Android fat percentage/gynoid fat percentage ratio (SDS)	0.96 (0.14–1.73)	*<0.001*	1.18 (0.34–1.84)	*<0.001*	0.69 (0.06–1.73)	*0.001*	*0.02*
TBLH BMC for age (SDS)	0.44 (–0.70 to 1.14)	0.16	0.17 (–0.42 to 1.19)	0.19	0.42 (–0.66 to 1.12)	0.16	0.80
TBLH BMC for bone area (SDS)	–1.29 (–2.22 to –0.45)	*<0.001*	–1.36 (–2.58 to –0.60)	*<0.001*	–1.52 (–2.15 to –0.56)	*<0.001*	0.57
TBLH bone area for height (SDS)	0.63 (–0.96 to 1.20)	0.12	0.85 (–0.51 to 1.74)	*0.02*	0.24 (–1.01 to 0.99)	0.54	0.35
TBLH BMD for age (SDS)	–0.41 (–1.45 to 0.48)	*0.01*	–0.45 (–1.80 to 0.51)	*0.008*	–0.22 (–1.23 to 0.51)	0.16	0.32

Data presented as median and interquartile ranges. For boys with multiple measurements, median SDS were used for comparison. *P*-values in italics are significant (*P* < 0.05).

^a^Comparison between patients and reference; ^b^comparison by multiple linear regression of patients before and during TRT (*n* = 17).

BMC, bone mineral content; BMD, bone mineral density; BMI, body mass index; DXA, dual-energy x-ray absorptiometry; SDS, standard deviation score; TBLH, total body less head; TRT, testosterone replacement therapy.

**Figure 3:**
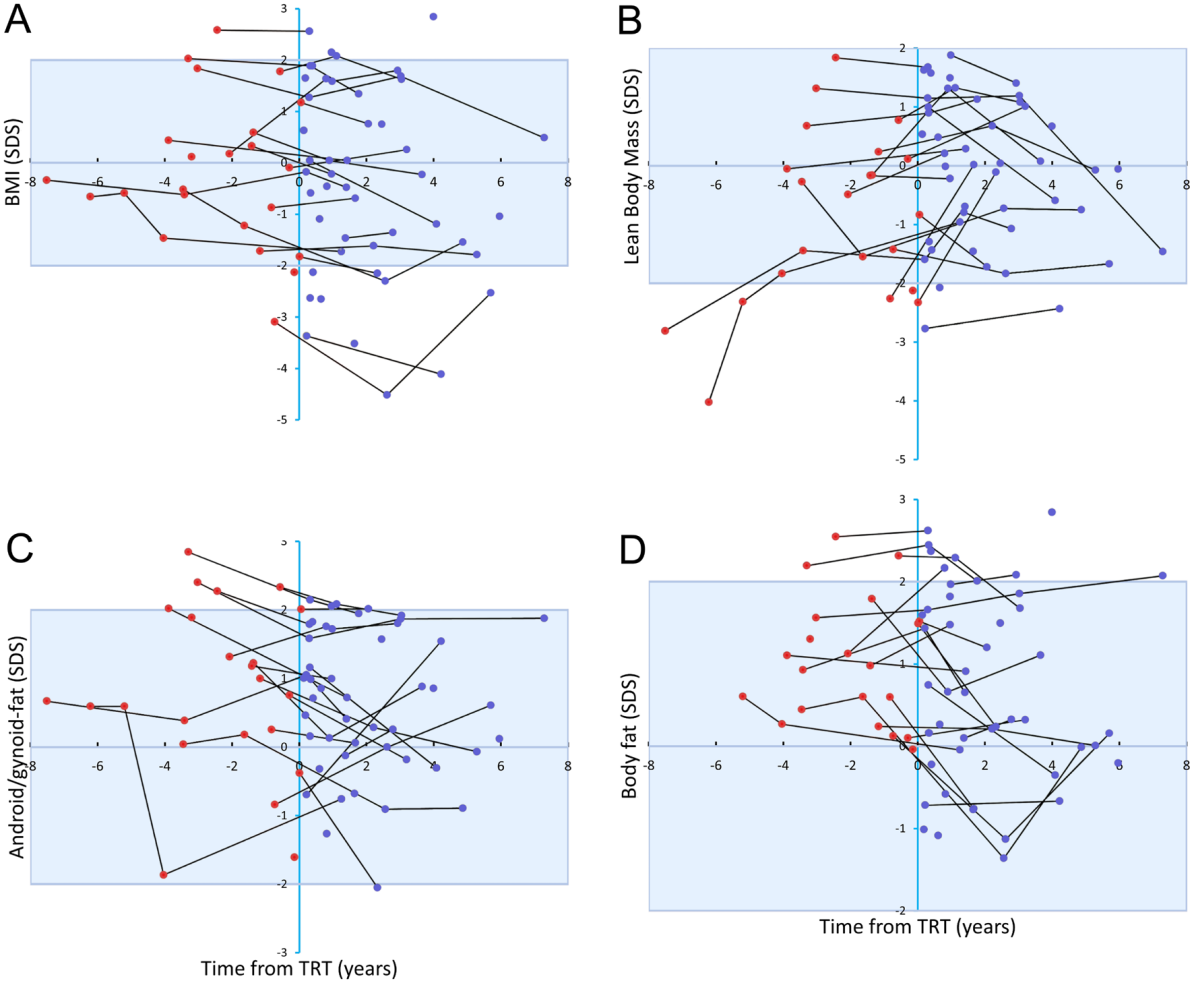
Age-adjusted BMI (A), lean body mass (B), ratio between android fat percentage and gynoid fat percentage (C), and body fat percentage (D) SDS according to the duration of testosterone replacement therapy in patients with Klinefelter syndrome. Longitudinal measures in the same individual are connected by a black line. Red dots indicate patients before testosterone replacement therapy, whereas blue dots indicate patients on testosterone replacement therapy. Blue lines and shading represent median and ±2 SDS for controls.

Body fat percentage, android fat percentage, gynoid fat percentage, as well as ratio between android fat percentage and gynoid fat percentage were significantly higher when compared to the reference (Table 1 and Fig. 2C, D, E and G). This result did not change when only analyzing patient data before TRT. Patients on TRT had normal gynoid fat percentage but significantly higher percentages of body fat, android fat, as well as ratio between android fat percentage and gynoid fat percentage when compared to the reference (Table 1). In patients before TRT, the lean body mass was lower, whereas no difference in lean body mass was found when comparing patients on TRT with the reference (Fig. 2B and Table 1). As seen in Fig. 3A, C and D, SDSs of BMI, ratio between android fat percentage and gynoid fat percentage as well as body fat percentage declined after starting TRT in most patients, whereas the changes in lean body mass were less clear (Fig. 3B). However, when comparing the results in patients evaluated both before and during TRT only the decline in the ratio between android fat percentage and gynoid fat percentage after TRT turned out significant (Table 1).

### Bone mineral content

BMC adjusted for bone area and BMD for age were lower when compared to the reference, but BMC for age and bone area adjusted for height did not differ (Table 1 and Fig. 1). The same result, except for a higher bone area adjusted for height, was found when only analyzing data on patients before TRT. In patients on TRT, only BMC for area was lower, whereas the other parameters did not differ from the reference. In Fig. 1F, BMD SDS is shown in relation to the timing of TRT. In some patients, there was a clear increase in BMD SDS after initiating TRT, but in others the opposite was observed. When comparing bone mineral status in patients before and during TRT (Table 1) and between prenatally and postnatally diagnosed patients (data not shown), no differences were found.

### Reproductive hormones

**Figure 4:**
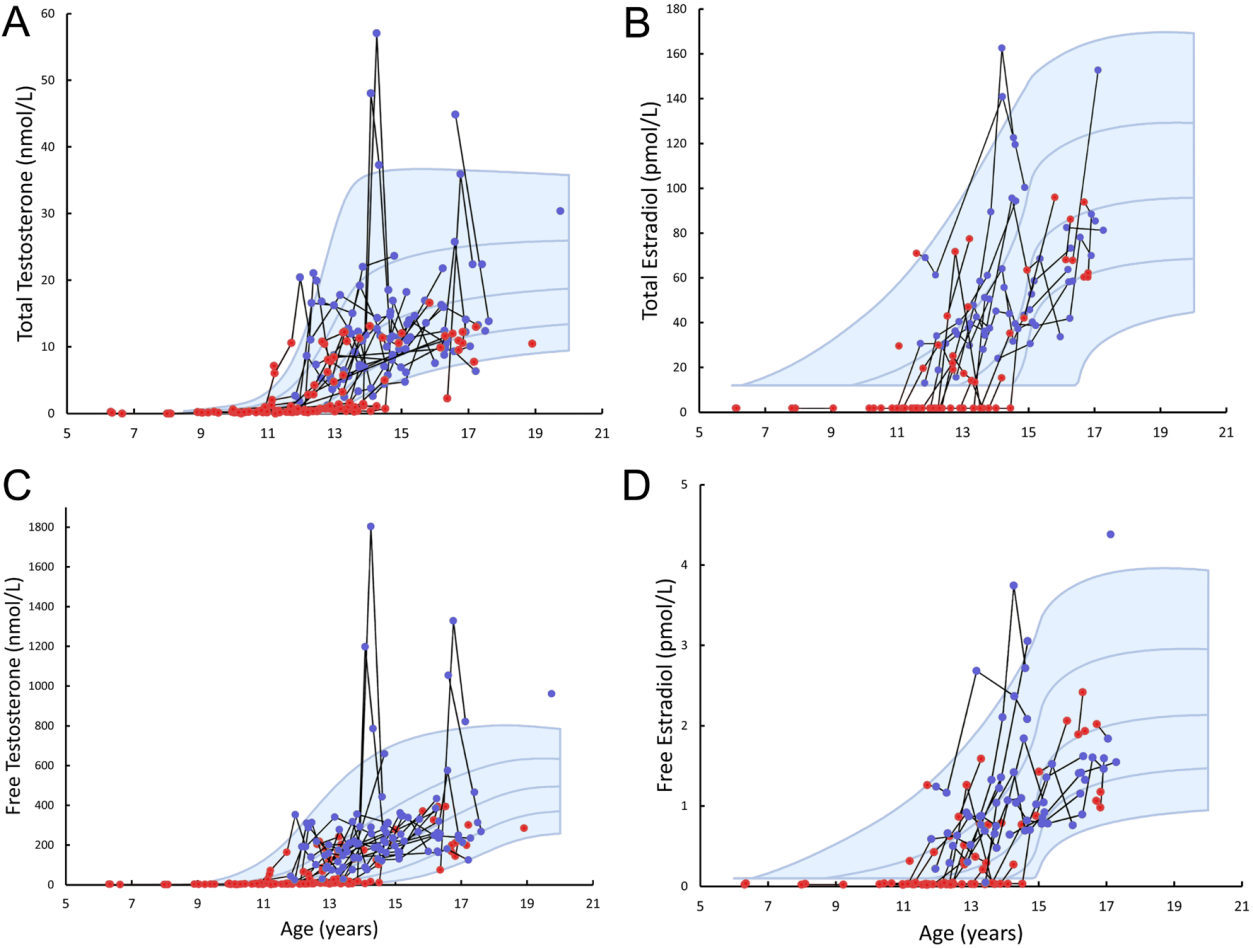
Serum concentrations of total testosterone (A), total estradiol (B), free testosterone (C), and free estradiol (D) according to age in patients with Klinefelter syndrome. Longitudinal measures in the same individual are connected by a black line. Red dots indicate patients before testosterone replacement therapy, whereas blue dots indicate patients on testosterone replacement therapy. Blue lines and shading represent median and ±1 SDS and ±2 SDS for controls.

**Figure 5:**
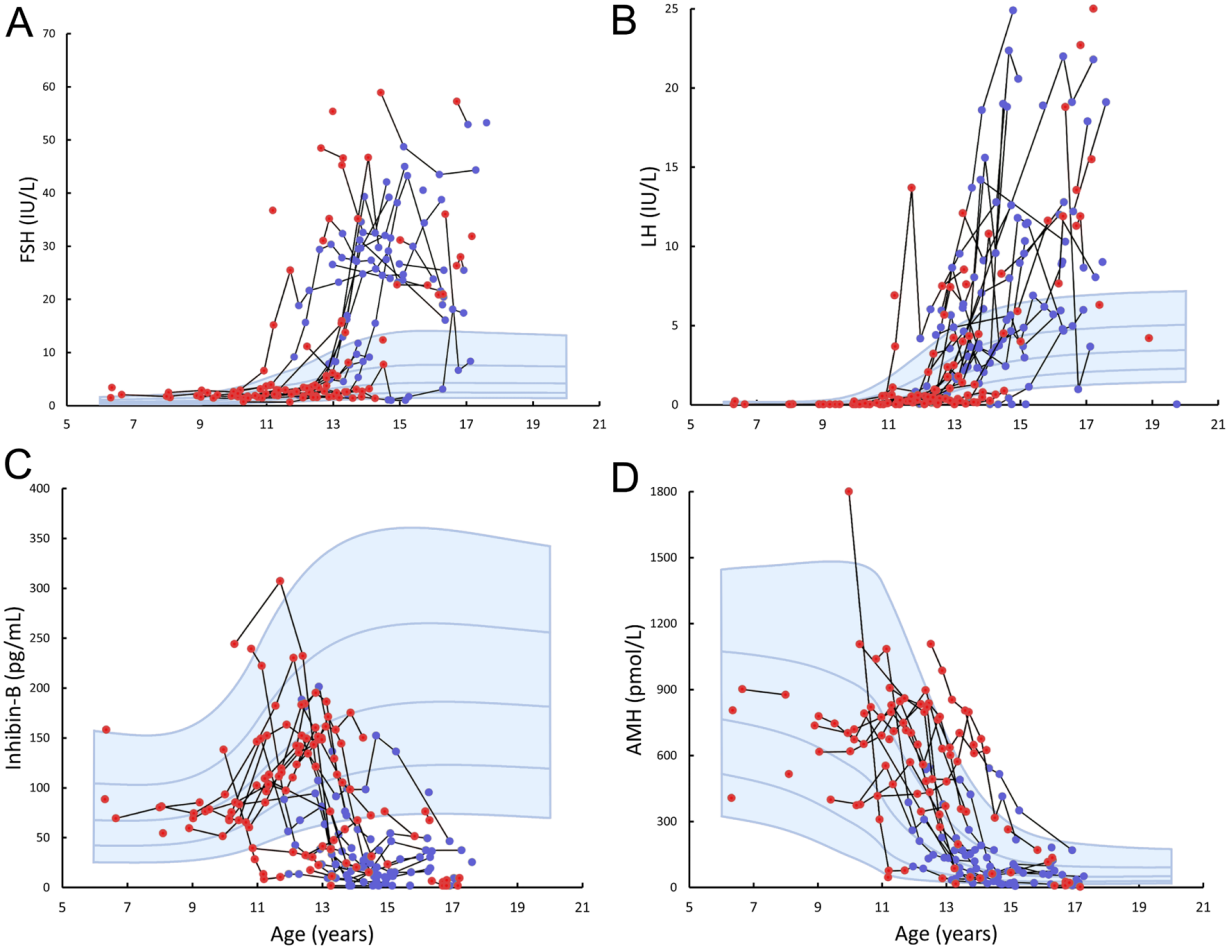
Serum concentrations of FSH (A), LH (B), inhibin B (C), and AMH (D) according to age in patients with Klinefelter syndrome. Longitudinal measures in the same individual are connected by a black line. Red dots indicate patients before testosterone replacement therapy, whereas blue dots indicate patients on testosterone replacement therapy. Blue lines and shading represent median and ±1 SDS and ±2 SDS for controls.

**Figure 6:**
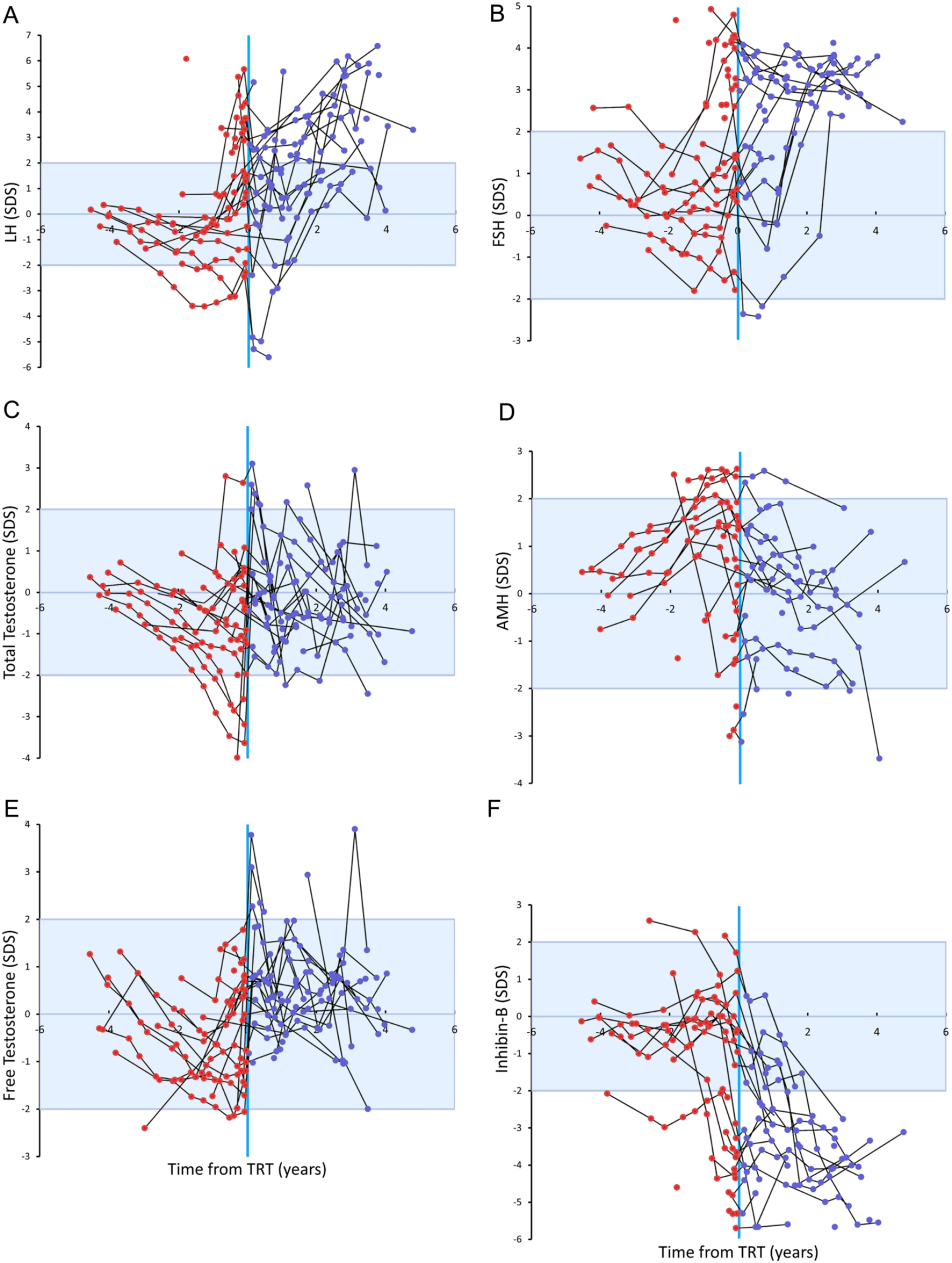
Age-adjusted standard deviation scores of serum concentrations of LH (A), FSH (B), total testosterone (C), AMH (D), free testosterone (E), and inhibin B (F) before and during testosterone replacement therapy in patients with Klinefelter syndrome. Longitudinal measures in the same individual are connected by a black line. Red dots indicate patients before testosterone replacement therapy, whereas blue dots indicate patients on testosterone replacement therapy. Blue lines and shading represent median and ±1 SDS and ±2 SDS for controls.

In patients before TRT, the serum concentrations of total testosterone and inhibin B were lower, and the concentrations of LH and FSH were higher when compared to the reference (Fig. 4A, 5A, B and C, and Table 2). Serum concentrations of free testosterone, total E2, free E2, and AMH did not differ from the reference (Fig. 4B, C and D, Fig. 5D, and Table 2). In patients on TRT, the serum concentrations of total testosterone, total E2, free E2, and AMH did not differ from the reference, whereas the concentration of inhibin B was lower, and the concentrations of free testosterone, LH and FSH concentrations were higher than the reference (Table 2). In the left panel of Fig. 6 (A, C and E) SDSs of LH, total testosterone, and free testosterone are illustrated before and during TRT. Before TRT, LH SDS (red dots) were within ±2 SDS in the majority of patients, and in most but not all patients LH SDS increased before start of the TRT (Fig. 6A). After initiating TRT, LH SDS (blue dots) declined followed by a steep increase in the majority (Fig. 6A). Before starting TRT, the SDS of total testosterone and free testosterone declined in the majority (Fig. 6C and E). In the right panel of Fig. 6 (B, D, and F), the SDSs of FSH, AMH, and inhibin B are shown. Like LH, FSH SDS declined after initiating TRT (Fig. 6B). The decline was followed by a steep increase (Fig. 6B) and FSH remained above +2 SDS in most. By contrast, no distinct changes were seen in AMH SDS before and during TRT (Fig. 6D), whereas inhibin B SDS declined before TRT (Fig. 6F) and continued to decline to far below –2 SDS in most patients (Fig. 6F).

### Testosterone replacement therapy

The patients started TRT at a median age of 13.9 years (10.6–20.2 years). The median duration of TRT at the latest DXA was 1.7 years (1 month–7.2 years). Thirteen patients were on TRT for less than 1 year at the latest DXA. In patients who were evaluated both before and during TRT (*n* = 17), the median duration of TRT at the latest DXA was 2.0 years (0.25–7.2 years).

**Figure 7:**
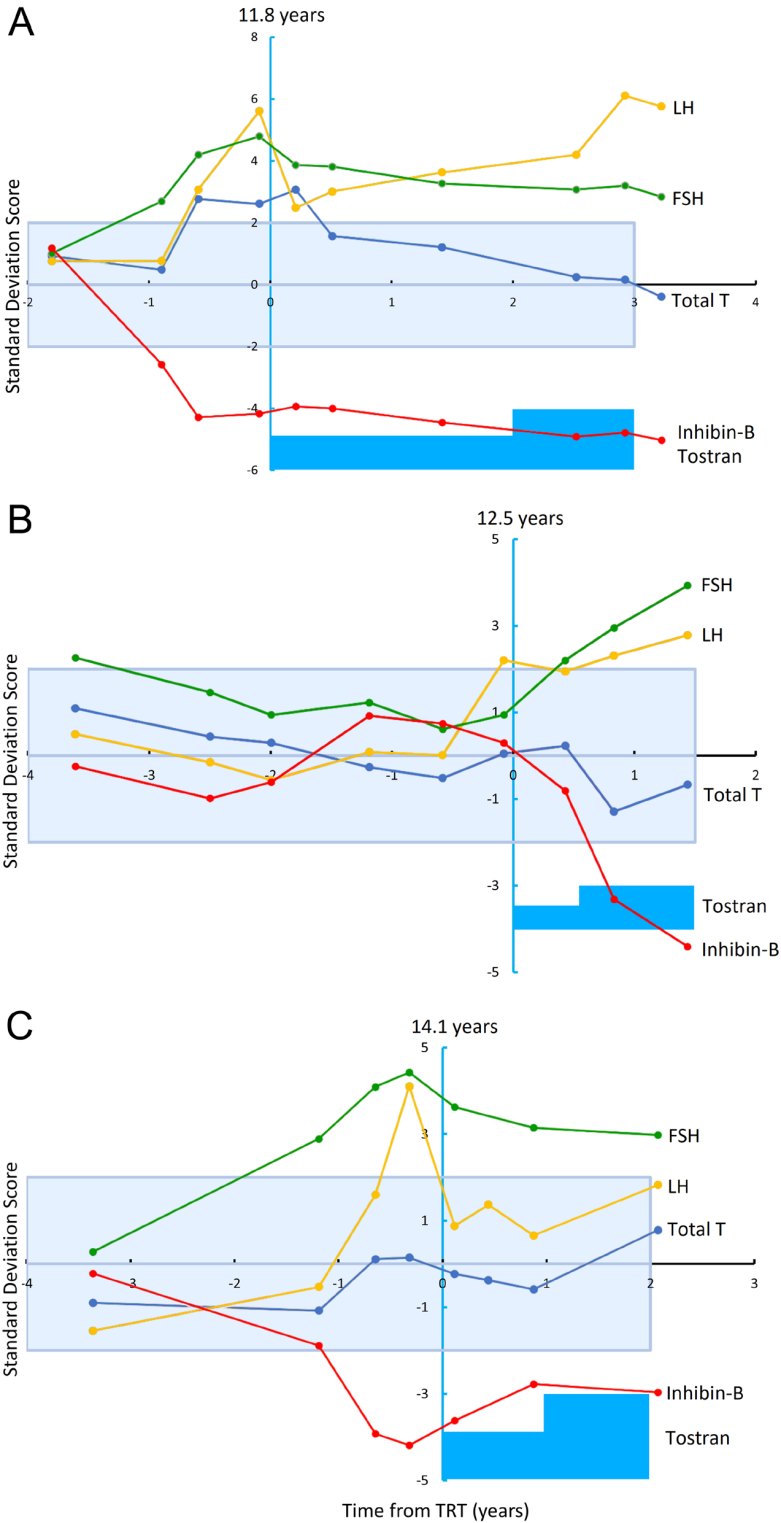
Age-adjusted standard deviation scores of longitudinal measurements of the serum concentration of LH, FSH, total testosterone, and inhibin B in three individuals with Klinefelter syndrome before and during testosterone replacement therapy. Yellow, green, blue, and red lines illustrate changes in the serum concentrations of LH, FSH, total testosterone, and inhibin B, respectively. Blue boxes illustrate the changes in the dose of transdermal testosterone. Patient A started on 10 mg transdermal testosterone every second day and patients B and C started on a daily dose of 10 mg transdermal testosterone. Dose was titrated according to the serum concentrations of testosterone.

In Fig. 7, the evolution in serum concentrations of total testosterone, LH, FSH, and inhibin B during pubertal initiation and dose titration of TRT from three individual patients is illustrated. The patient in Fig. 6A started TRT at the age of 11.8 years, whereas the other patients started at the age of 12.5 and 14.1 years (Fig. 7B and C, respectively). In two out of the three patients (Fig. 7A and C), a transient decline followed by an increase in LH was observed after initiating TRT. The illustrated patients all started on transdermal testosterone with either 10 mg applied every second day (Fig. 7A) or every day (Fig. 7B and C).

## Discussion

We present longitudinal data on anthropometrics, serum concentrations of reproductive hormones, as well as body composition and BMC during pubertal transition in 62 boys and adolescents with KS in relation to age-adjusted references based on 2823 healthy Danish boys and adolescents aged 7.7–20.0 years.

The patients presented with tall stature, low total testosterone and inhibin B, and elevated concentrations of LH and FSH before TRT. Body fat percentage and ratio between android fat percentage and gynoid fat percentage were within the normal range but significantly higher when compared to the reference irrespective of treatment status. Lean body mass was low only in untreated patients, but in patients evaluated before and during TRT, a tendency toward a more beneficial body composition with a significant reduction in the ratio between android fat percentage and gynoid fat percentage during TRT was found. BMC (BMC for age) was normal, but BMC adjusted for bone size (BMC for bone area) was significantly lower than the reference irrespective of the treatment status. These results confirm that patients with KS appear to have an unfavorable body composition and impaired bone status already during childhood and adolescence.

### Body composition

It is well described that adult patients with KS have an unfavorable metabolic phenotype, including truncal obesity, insulin resistance, and type II diabetes (6, 26, 27). Epidemiological studies have shown an increased risk of dying from diabetes or being hospitalized with diabetes (28, 29). For any given BMI, men with KS have higher truncal fat percentage as compared with controls, and truncal adiposity has been shown to be the major predictor of metabolic syndrome and insulin sensitivity in these patients (27). In previous studies, an unfavorable body composition with truncal obesity, insulin resistance, and metabolic syndrome already during childhood and adolescence in patients with KS has been shown (9, 10, 11, 30). Bardsley *et al.* found increased waist circumference >90 percentile in 30% of boys with KS (*n* = 89), and 24% of these boys had insulin resistance, whereas 7% met the criteria for metabolic syndrome (10). In line with this, Davis *et al.* studied 93 boys with KS aged 4.0–12.9 years and found a waist-to-hip ratio (as a measure of abdominal adiposity) above the 95th percentile in 45% of the patients. Seventy-nine percent of the patients presented with at least one feature of metabolic syndrome (waist circumference, fasting lipid panel, fasting blood glucose, and blood pressure), while 11% met the criteria for metabolic syndrome (9). In that study, BMI was normal, and no association between BMI and the metabolic profile was found (9). These findings are in accordance with a study on obese children aged 6–17 years, where an increased ratio between android fat percentage and gynoid fat percentage was associated with insulin resistance, but no association between insulin resistance and BMI or body fat percentage was found (31). These studies indicate that truncal adiposity is associated with increased risk of developing insulin resistance already in childhood and adolescence. We do not have data on metabolic parameters in our study; however, our finding of normal BMI but significantly increased body fat percentage and specifically increased ratio between android fat percentage and gynoid fat percentage indicates that the patients in the current study may be at increased risk of having an impaired metabolic profile.

Testosterone deficiency is associated with adiposity and insulin resistance in men without chromosome aberrations, but the relationship is complex and bidirectional (32). In patients with KS, testosterone deficiency is a common finding, and the classical adult with KS presents with low to low-normal serum concentrations of testosterone in combination with highly elevated LH and FSH (3, 27, 33). Bojesen *et al.* found a significant association between serum concentrations of total testosterone and insulin sensitivity and the presence of metabolic syndrome in adults with KS, but the association disappeared when correcting for truncal fat percentage (27). In a recent meta-analysis of studies on adults with KS it was shown that TRT improved the body composition by a reduction in fat mass and increase in lean mass, but no effect on BMI, waist circumference, or metabolic profile was found (34).

In the study by Davis *et al.,* an impaired Sertoli cell function with reduced serum concentrations of inhibin B and AMH was associated with increased cardiometabolic risk (9). However, there was no association between the cardiometabolic profile and total testosterone despite a significantly reduced serum concentration of total testosterone (9). We found significantly reduced serum concentration of total testosterone, inhibin B, and AMH combined with significantly increased serum concentrations of LH and FSH in the untreated patients with KS. Our patients on TRT had normal serum concentrations of total testosterone but still elevated LH and FSH.

In a double-blind, randomized trial, Davis *et al.* showed that a 2-year treatment with oxandrolone (a synthetic nonaromatizable testosterone derivative) in prepubertal boys with KS nearly normalized body fat percentage, but it did not affect metabolic parameters except a reduction in triglycerides as well as an unwanted reduction in HDL (35). It was noted that the effects on body composition only appeared in the second year of treatment, and it may therefore be speculated that a longer duration of treatment may have beneficial effects on metabolic parameters. Our study was retrospective and observational and, thus, not designed to evaluate the effect of TRT on body composition. However, androgens are known to improve the body composition by increasing the lean body mass and decreasing the body fat percentage (36), and our finding of an improved body composition with higher lean body mass and lower ratio between android fat percentage and gynoid fat percentage in patients during TRT compared to before TRT confirmed this.

### Bone mineral content

Lower BMD and altered bone microarchitecture has been reported in studies of adults with KS (26, 37, 38, 39, 40, 41, 42). In a recent meta-analysis of 1141 patients with KS, a reduced BMD with an improvement after initiation of TRT was observed (34), but the relation between testosterone and bone mineral status in KS is not completely clear. In epidemiological studies, an increased risk of osteoporosis-related (spine, hip, and forearm) fractures (28, 29) has been reported, and in a recent study on 87 adults with KS by Vena *et al.,* a high prevalence of symptomatic vertebral fractures independent of BMD was found (40). Thus, an impaired BMC poses a significant risk to the health and quality of life in adult patients with KS.

Only few studies of BMC in children and adolescents with KS exist (11, 12, 13). Stagi *et al.* evaluated bone mineral status expressed as amplitude-dependent speed of sound and bone transmission time in 40 boys with KS evaluated by quantitative ultrasound scan (13). The authors found significantly reduced amplitude-dependent speed of sound and bone transmission time SDS when compared with healthy subjects, and in more than 17% of the patients SDSs were below –2.0 (13).

In the previously mentioned, double-blind, randomized trial on oxandrolone in 89 prepubertal boys with KS the bone health index (BHI) was calculated from left-hand radiographs (12). At baseline, BHI SDSs were significantly reduced in the patients compared to controls (12). After 2 years of follow-up, BHI declined further in the placebo group, whereas treatment with oxandrolone increased BHI. Optimal bone mineralization during childhood and adolescence is dependent on normal pubertal development, and impaired androgen concentration at this critical stage of bone maturation may affect peak bone mass acquisition (43). The finding of a decline in BHI during follow-up in the placebo group may indicate that bone mass acquisition is impaired in boys with KS.

Previously, we found no difference in whole-body BMC or BMD in 24 patients with KS aged 4.3–18.6 years (untreated; *n* = 18) compared to the reference (11). In the current study, we found no difference in BMC for age (whole group and irrespective of treatment status) but a reduced BMD for age in the whole group and in untreated patients compared to the reference was observed. Interestingly, BMC adjusted for bone area was significantly reduced, indicating that the patients in the current study had ‘light bones’. Thus, despite a normal BMC for age, BMC was in fact impaired when adjusting for bone size. This finding was independent of treatment status. As the patients in our study were significantly taller than controls, adjusting BMC for bone size was important (44). Surprisingly, bone area adjusted for height was increased in patients before TRT, whereas no differences were found when analyzing the whole group or patients during TRT. This may indicate that these patients have ‘wide bones’, although the opposite would be expected since patients with KS are tall with accelerated growth before puberty (45).

### TRT during puberty

It has generally been recommended to initiate TRT early during puberty (46, 47, 48), and the treatment is well tolerated (49, 50). However, there are no evidence-based recommendations concerning when or how to start TRT in KS, and recently this clinical practice has been questioned (51). In our department, treatment with testosterone is typically initiated when LH exceeds +2 SDS or if the patient has symptoms or develops phenotypic traits associated with testosterone deficiency (e.g. gynecomastia, increased fat percentage, and diminished virilization). The majority are treated with transdermal testosterone, with dose titration according to the serum concentrations of testosterone, whereas gonadotropins are not normalized. In line with a study on Danish boys with delayed puberty, we found a transient suppression in the concentrations of LH and FSH following initiation of TRT in a subgroup of patients. It has been speculated that such suppression may represent differences in the sensitivity to exogenous testosterone of the pituitary–gonadal axis, but the clinical implication remains unknown (52).

Although a clear benefit of TRT in most adult patients regarding social and sexual functioning can be seen, it is not well understood how TRT affects the general morbidity and mortality. It has been discussed whether early treatment with testosterone during puberty may improve bone mineral accrual and body composition and thereby potentially reduce the future risk of osteoporosis, insulin resistance, and metabolic syndrome (53, 54, 55, 56, 57). So far, no randomized, placebo-controlled trials on TRT in pubertal boys with KS exist. The study by Davis *et al.* included prepubertal boys aged 4.0–12.9 years with KS who were randomized to a 2-year treatment with either low-dose oral oxandrolone or placebo. The authors reported improvements in bone mass, body composition, fasting triglycerides, measures of anxiety, and visual-motor integration compared with placebo but no significant changes in cognition or motor function (12, 35, 58). However, the patients on oxandrolone developed growth acceleration, and gonadarche was reached 2 years earlier than in the placebo group.

### Strengths and limitations

The major strength of this study is the well-characterized cohort of boys and adolescents with KS and the inclusion of a large cohort of 2823 healthy controls for the development of new references on DXA-derived evaluation of body composition and BMC in boys and adolescents. Importantly, all patients and controls were scanned with the same type of DXA scan machine using the same software in the same department. Further, all reproductive hormones of both patients and references were determined by highly sensitive methods in our own laboratory. Another important strength is the calculation of SDSs for all parameters (anthropometrics, DXA-derived parameters, and hormone concentrations) allowing for comparison across ages. The study also has limitations, which need to be addressed. Some, but not all, patients had repeated DXA scans and evaluations of reproductive hormones, but some patients did not have a complete set of reproductive hormones. The study was retrospective and not designed to evaluate the effect of TRT, and the design did not allow for taking the timing of blood sampling in relation to the time of applying TRT into account. A weakness of the reference material is that there are no controls between ages 14 and 18, and GAMLSS calculation for this age interval was done by interpolation. However, we do not expect dramatical fluctuations in the evaluated parameters between 14 and 18 years, and since we have values both before and after the missing values, we were able to see that values of the controls aged 18–20 years came as a natural prolongation of the childhood values.

## Conclusion

Our study confirms previous studies that children and adolescents with KS have an unfavorable body composition with truncal obesity despite normal BMI. Importantly, our results indicate that these patients also have an impaired bone mineral status when adjusting BMC for bone size. In patients evaluated before and during TRT, the ratio between android fat percentage and gynoid fat percentage declined during TRT. However, this result must be interpreted with caution since the study was not designed to evaluate the effect of TRT on bone or body composition in these patients. Previous studies indicate that these phenotypic features may be directly or indirectly attributed to testosterone deficiency, but growing evidence support that other factors such as changes to the epigenome and transcriptome also play an important role and need to be investigated further.

Our findings emphasize the need for further studies on the natural history of the adverse bone mineral status and body composition in KS and on the effect of early TRT in puberty in these patients. Our findings also underline the importance of thorough clinical evaluations of the child with KS and guidance regardless of BMI.

## Supplementary Materials

Supplementary Table 1

Supplementary Figure 1 Body fat% (A), lean body mass (B), and ratio between android fat% and gynoid fat% (C) according to age in controls. Blue dots indicate individual measurements of the controls. Blue lines and shading represent median, ±1SDS, and ± 2SDS for the controls.

Supplementary Figure 2 Bone mineral content (BMC) (A) and bone mineral density (BMD) (B) according to age in controls. Blue dots indicate individual measurements of the healthy controls. Blue lines and shading represent median, ±1SDS, and ± 2SDS for the controls. Blue lines and shading represent median, ±1SDS, and ± 2SDS for the controls.

Supplementary Figure 3 Bone mineral content (BMC) according to bone area (A) and bone area according to height (B) in controls. Blue dots indicate individual measurements of the healthy controls. Blue lines and shading represent median, ±1SDS, and ± 2SDS for the controls. Blue lines and shading represent median, ±1SDS, and ± 2SDS for the controls.

## Declaration of interest

The authors declare that there is no conflict of interest that could be perceived as prejudicing the impartiality of the research reported.

## Funding

This study was supported by Rigshospitalethttp://dx.doi.org/10.13039/501100005111’s Research council (AJ and LA).
