# Supplementary material for: Reproductive hormones, bone mineral content, body composition, and testosterone therapy in boys and adolescents with Klinefelter syndrome
Source: Endocr Connect. 2023 Jun 12;12(7):e230031. doi: 10.1530/EC-23-0031 (PMC10305500; doi:10.1530/EC-23-0031)
Supplement: Supplementary Table 1 [file supplementary_table_1.pdf]

Supplementary Table 1

Selected age-specific M, L, and S for Body Fat%, Lean Body Mass and Android/Gynoid fat-ratio in controls

Age (years)	Body Fat%			Lean Body Mass (g)			Android/Gynoid fat-ratio		
	M	L	S	M	L	S	M	L	S
**7.5**	0.169	0.086	0.452	19799.38	0.208	0.092	0.496	0.633	0.255
**8.0**	0.169	0.087	0.446	20846.15	0.116	0.096	0.507	0.562	0.258
**8.5**	0.170	0.088	0.441	21894.14	0.024	0.100	0.519	0.492	0.261
**9.0**	0.170	0.089	0.434	22956.94	-0.068	0.105	0.531	0.422	0.263
**9.5**	0.171	0.090	0.427	24042.19	-0.160	0.110	0.545	0.350	0.266
**10.0**	0.172	0.091	0.419	25144.05	-0.251	0.115	0.559	0.279	0.270
**10.5**	0.173	0.092	0.410	26274.92	-0.337	0.120	0.571	0.211	0.273
**11.0**	0.174	0.090	0.402	27483.61	-0.417	0.125	0.580	0.149	0.277
**11.5**	0.176	0.086	0.395	28820.67	-0.488	0.130	0.587	0.094	0.280
**12.0**	0.178	0.079	0.388	30324.39	-0.544	0.135	0.596	0.046	0.282
**12.5**	0.180	0.068	0.381	32018.89	-0.584	0.139	0.608	0.0062	0.282
**13.0**	0.182	0.052	0.374	33883.73	-0.603	0.142	0.622	-0.022	0.281
**13.5**	0.185	0.032	0.365	35891.71	-0.560	0.144	0.639	-0.040	0.279
**14.0**	0.187	0.0074	0.357	38008.96	-0.574	0.145	0.658	-0.046	0.276
**14.5**	0.190	-0.021	0.348	40188.09	-0.527	0.144	0.678	-0.043	0.273
**15.0**	0.192	-0.053	0.338	42390.41	-0.462	0.142	0.699	-0.033	0.268
**15.5**	0.195	-0.087	0.329	44571.52	-0.380	0.139	0.721	-0.018	0.264
**16.0**	0.197	-0.124	0.319	46684.93	-0.282	0.135	0.742	0.00053	0.259
**16.5**	0.199	-0.162	0.310	48684.14	-0.168	0.132	0.762	0.020	0.253
**17.0**	0.200	-0.200	0.302	50522.59	-0.041	0.128	0.780	0.037	0.247
**17.5**	0.202	-0.239	0.293	52153.76	0.098	0.125	0.795	0.051	0.241
**18.0**	0.202	-0.278	0.285	53531.61	0.248	0.122	0.808	0.060	0.235
**18.5**	0.203	-0.315	0.277	54618.71	0.407	0.120	0.817	0.060	0.228
**19.0**	0.202	-0.351	0.271	55406.72	0.572	0.119	0.823	0.051	0.222
**19.5**	0.202	-0.385	0.264	56016.95	0.736	0.120	0.829	0.037	0.216
**20.0**	0.201	-0.419	0.258	56627.67	0.896	0.121	0.836	0.020	0.211

Supplementary Table 2
Selected age-specific M, L, and S for BMC and BMD in controls

Age (years)	BMC (g)			BMD (g/cm^2)		
	M	L	S	M	L	S
**7.5**	626.959	0.418	0.138	0.709	1.951	0.058
**8.0**	686.218	0.342	0.145	0.725	1.706	0.060
**8.5**	747.602	0.267	0.153	0.742	1.462	0.062
**9.0**	811.103	0.191	0.1601	0.758	1.205	0.064
**9.5**	873.272	0.122	0.168	0.775	0.908	0.066
**10.0**	932.634	0.087	0.175	0.791	0.651	0.067
**10.5**	996.022	0.076	0.182	0.807	0.504	0.069
**11.0**	1068.404	0.046	0.188	0.824	0.412	0.071
**11.5**	1145.967	-0.010	0.195	0.842	0.345	0.074
**12.0**	1234.232	-0.086	0.201	0.863	0.298	0.079
**12.5**	1331.826	-0.145	0.206	0.886	0.286	0.084
**13.0**	1438.326	-0.167	0.208	0.912	0.293	0.090
**13.5**	1556.729	-0.170	0.206	0.939	0.265	0.095
**14.0**	1683.343	-0.155	0.201	0.968	0.248	0.098
**14.5**	1815.131	-0.120	0.192	0.997	0.259	0.099
**15.0**	1947.971	-0.068	0.182	1.026	0.294	0.100
**15.5**	2077.109	-0.0037	0.172	1.054	0.346	0.100
**16.0**	2197.767	0.069	0.162	1.082	0.410	0.099
**16.5**	2305.166	0.144	0.153	1.107	0.479	0.098
**17.0**	2394.529	0.218	0.145	1.129	0.547	0.098
**17.5**	2461.133	0.287	0.140	1.148	0.608	0.097
**18.0**	2501.333	0.347	0.137	1.163	0.658	0.098
**18.5**	2514.99	0.395	0.137	1.174	0.674	0.099
**19.0**	2518.252	0.445	0.139	1.182	0.635	0.101
**19.5**	2538.58	0.487	0.138	1.190	0.535	0.101
**20.0**	2563.209	0.533	0.137	1.197	0.428	0.101

Supplementary Table 3
Selected age-specific M, L, and S for BMC for Area and Area for Height in controls

Area (cm^2)	BMC (g)			Height (cm)	Area (cm^2)		
	M	L	S		M	L	S
700	459.313	6.947	0.033	120	839.344	-4.857	0.072
780	532.548	6.192	0.036	123	867.935	-4.462	0.071
860	606.,438	5.437	0.038	126	898.592	-4.038	0.070
940	685.489	4.684	0.041	129	932.138	-3.574	0.070
1020	764.046	3.929	0.044	132	970.293	-3.047	0.069
1100	844.152	3.131	0.047	135	1019.261	-2.376	0.068
1180	932.051	2.228	0.049	138	1081.335	-1.557	0.066
1260	1025.454	1.253	0.051	141	1143.239	-0.817	0.065
1340	1117.556	0.317	0.053	144	1206.074	-0.187	0.065
1420	1212.966	-0.485	0.057	147	1271.114	0.306	0.064
1500	1315.23	-1.059	0.063	150	1339.432	0.660	0.063
1580	1436.67	-1.328	0.071	153	1411.754	0.885	0.063
1660	1584.053	-1.260	0.079	156	1488.419	0.990	0.062
1740	1755.322	-0.941	0.087	159	1568.964	0.972	0.061
1820	1935.892	-0.488	0.094	162	1651.98	0.836	0.060
1900	2109.027	-0.029	0.097	165	1735.221	0.604	0.058
1980	2269.831	0.321	0.099	168	1816.415	0.304	0.057
2060	2414.355	0.533	0.099	171	1893.505	-0.0064	0.055
2140	2542.532	0.647	0.098	174	1964.981	-0.254	0.053
2220	2662.143	0.681	0.096	177	2030.341	-0.395	0.052
2300	2782.009	0.706	0.096	180	2089.998	-0.415	0.050
2380	2907.934	0.774	0.095	183	2144.482	-0.323	0.049
2460	3034.933	0.954	0.095	186	2194.997	-0.155	0.049
2540	3164.349	1.232	0.095	189	2243.182	0.023	0.049
2620	3295.215	1.560	0.095	192	2290.424	0.169	0.048
2700	3426.837	1.902	0.095	195	2337.596	0.290	0.049
2780	3558.864	2.248	0.095	198	2384.838	0.396	0.049
2860	3690.936	2.594	0.095	201	2432.137	0.491	0.049
2940	3823.008	2.941	0.095	204	2479.452	0.582	0.050
