# Supplementary figures and images for: Reproductive hormones, bone mineral content, body composition, and testosterone therapy in boys and adolescents with Klinefelter syndrome
Source: Endocr Connect. 2023 Jun 12;12(7):e230031. doi: 10.1530/EC-23-0031 (PMC10305500; doi:10.1530/EC-23-0031)

Figure 1

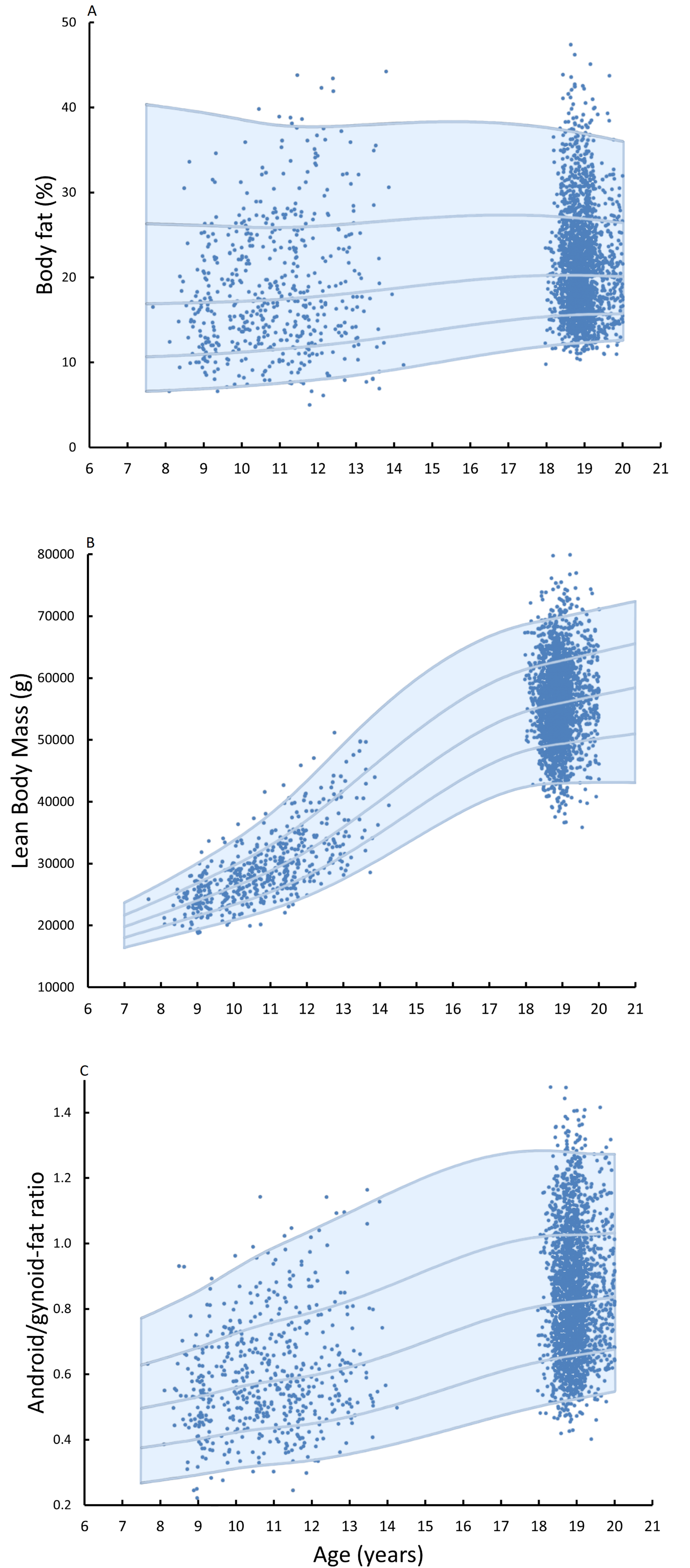

Supplement: Supplementary Figure 1 Body fat% (A), lean body mass (B), and ratio between android fat% and gynoid fat% (C) according to age in controls. Blue dots indicate individual measurements of the controls. Blue lines and shading represent median, ±1SDS, and ± 2SDS for the controls. [file supplementary_figure_1.pdf]

Figure 2

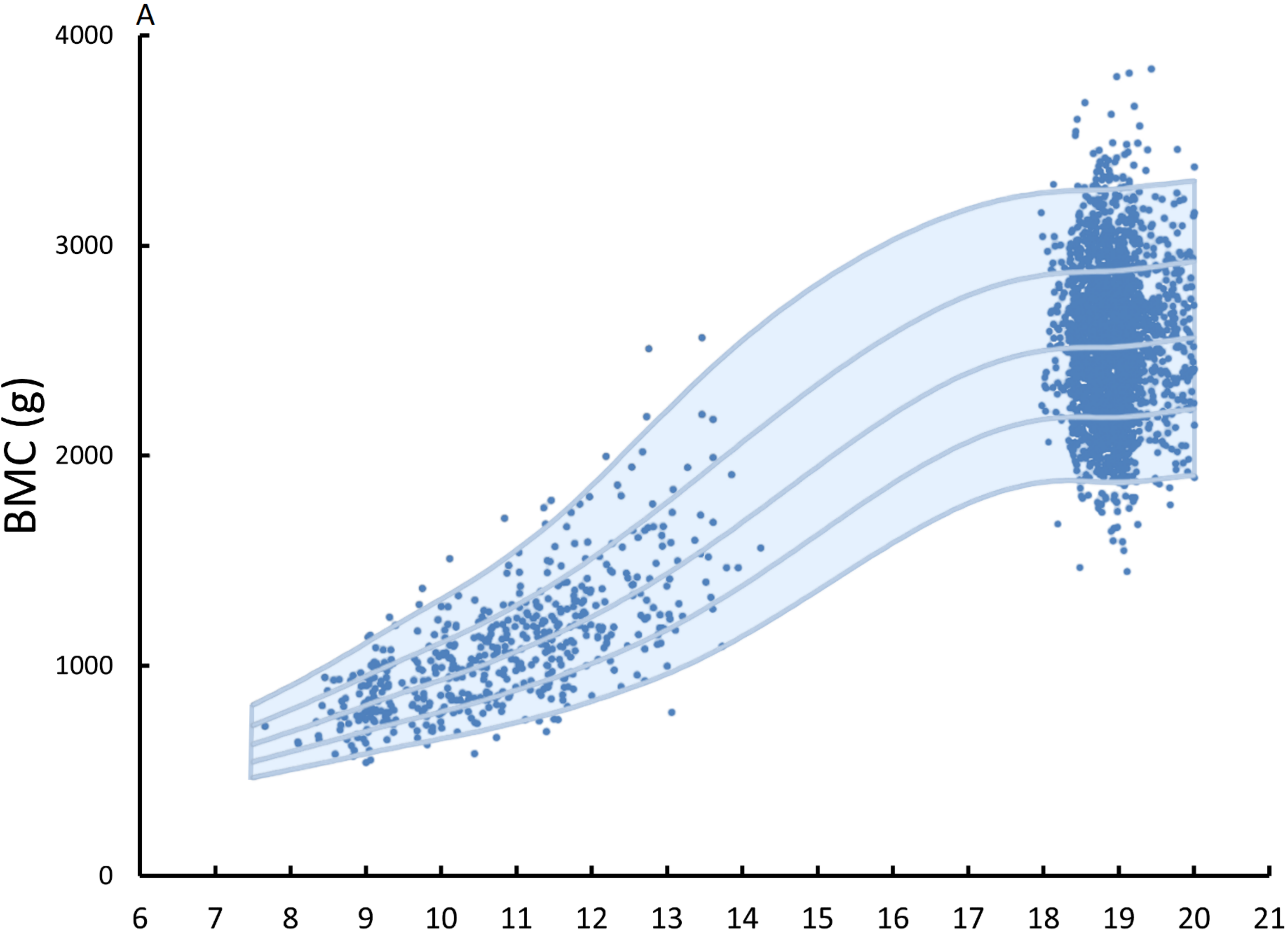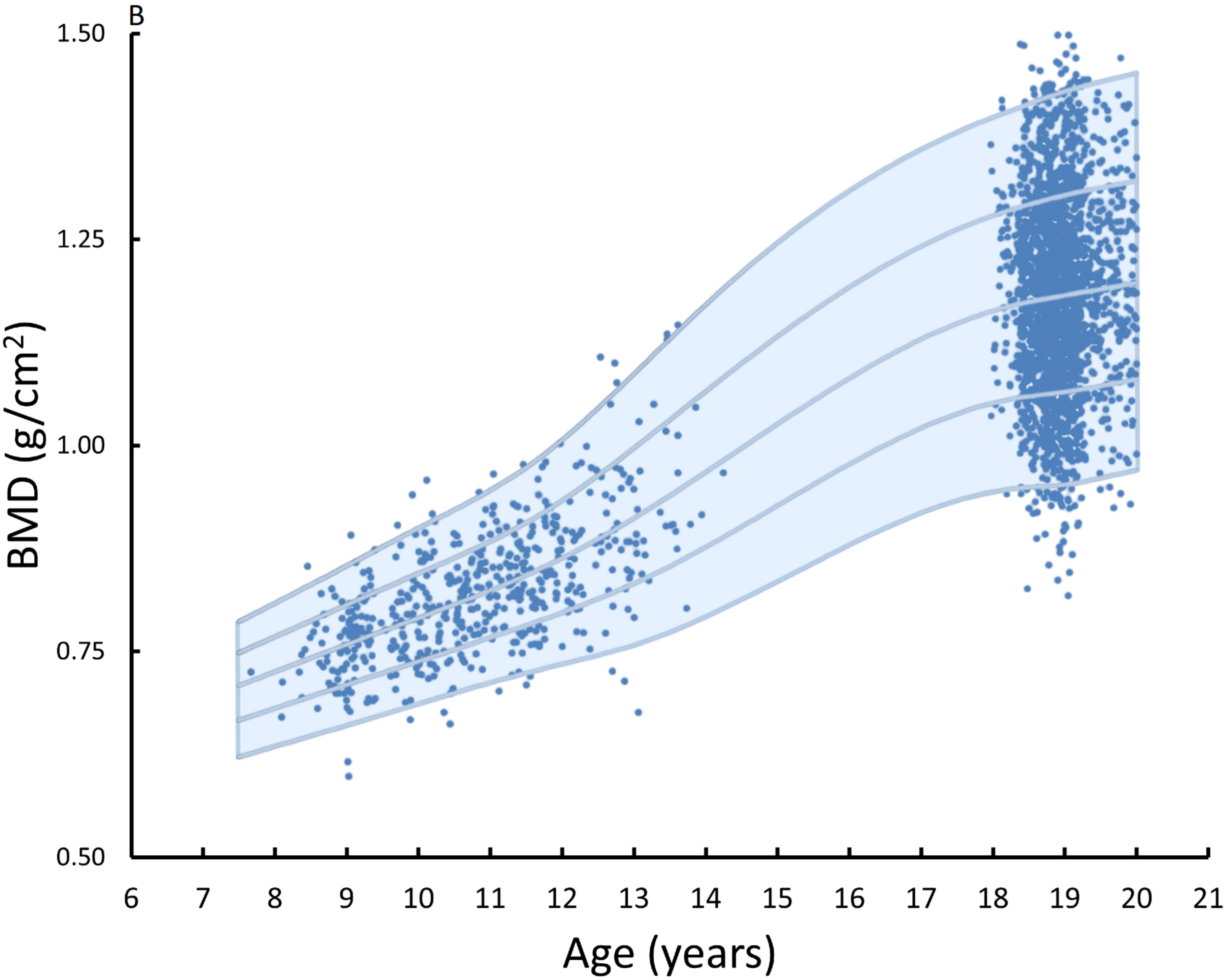

Supplement: Supplementary Figure 2 Bone mineral content (BMC) (A) and bone mineral density (BMD) (B) according to age in controls. Blue dots indicate individual measurements of the healthy controls. Blue lines and shading represent median, ±1SDS, and ± 2SDS for the controls. Blue lines and shading represent med [file supplementary_figure_2.pdf]

Figure 3

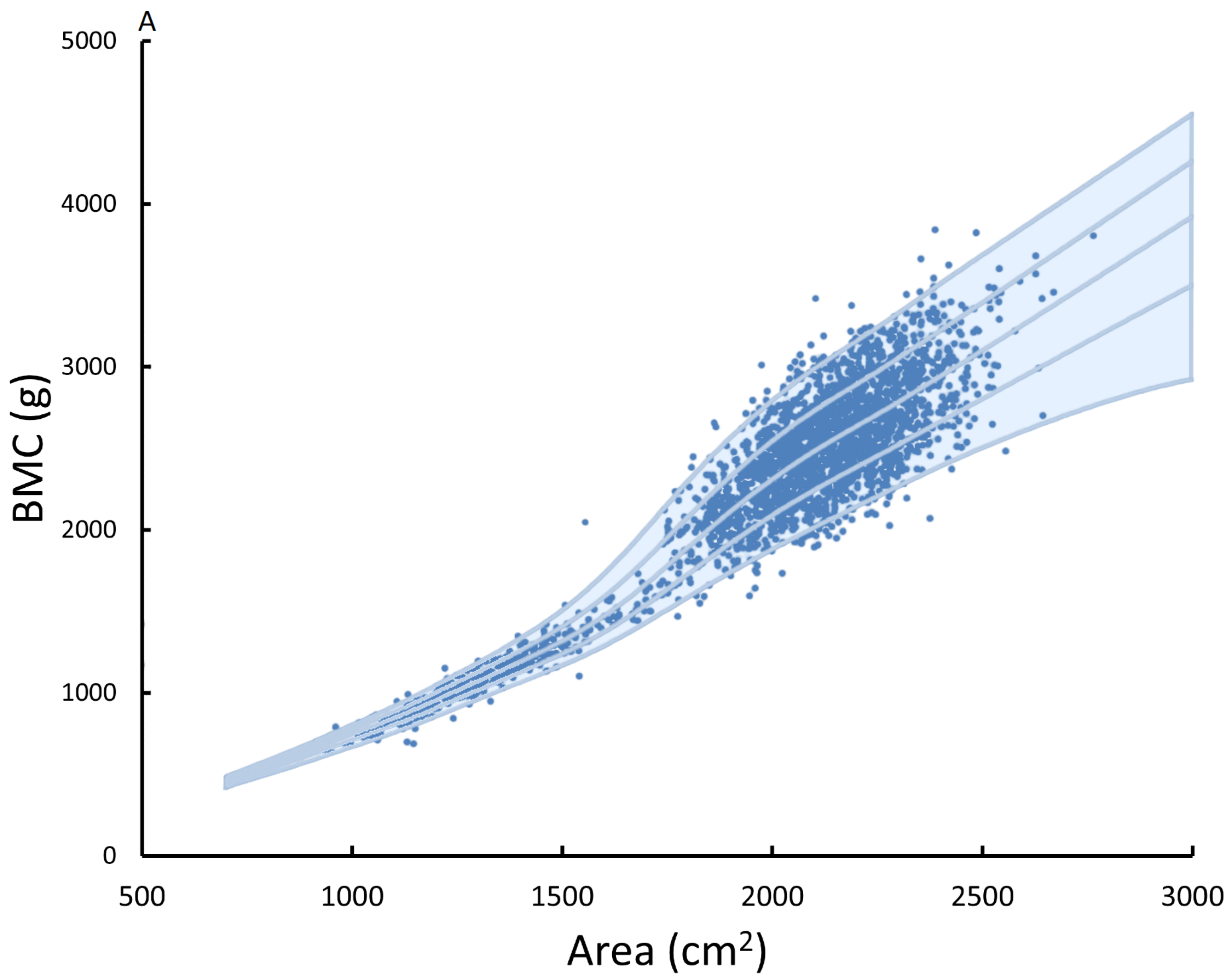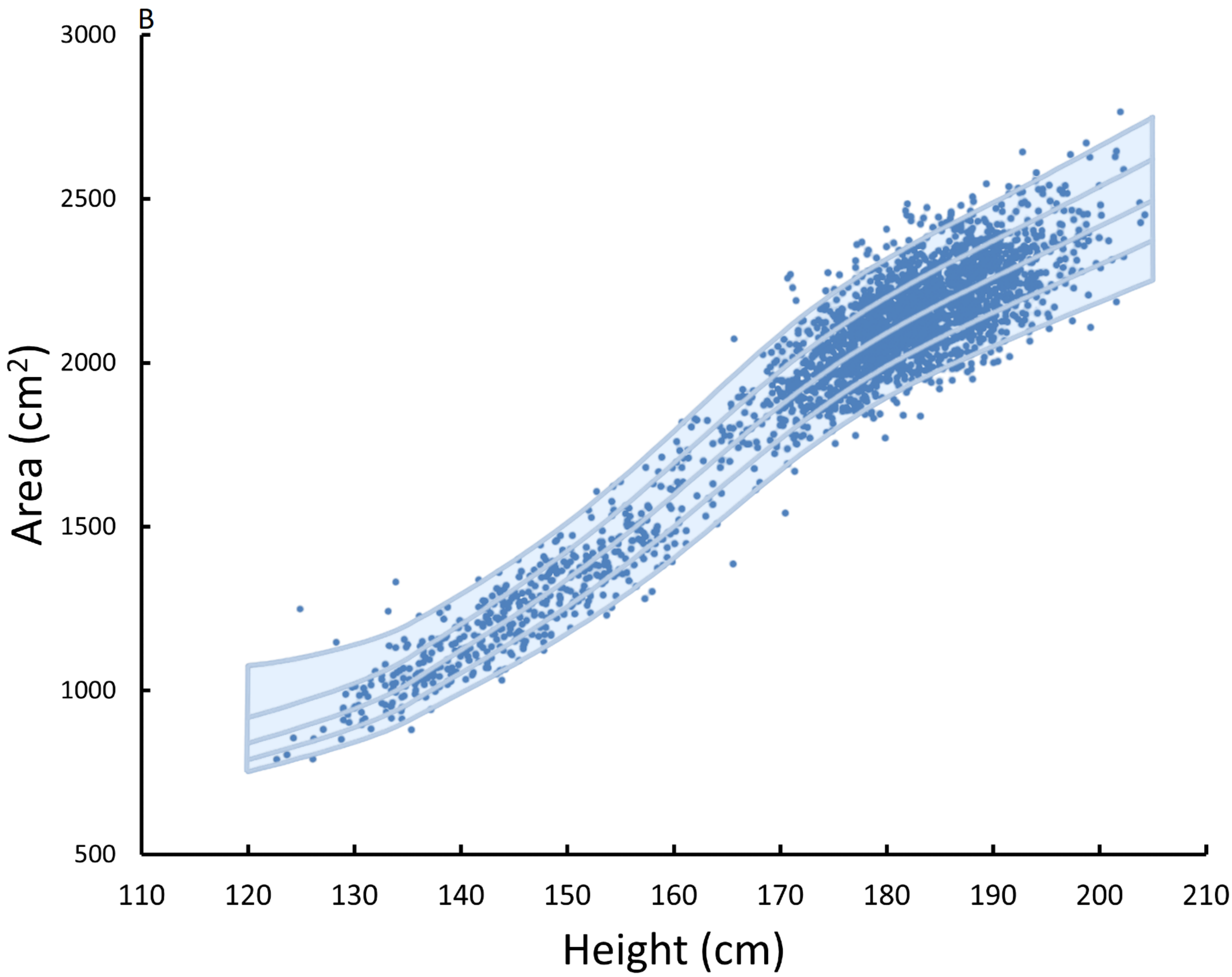

Supplement: Supplementary Figure 3 Bone mineral content (BMC) according to bone area (A) and bone area according to height (B) in controls. Blue dots indicate individual measurements of the healthy controls. Blue lines and shading represent median, ±1SDS, and ± 2SDS for the controls. Blue lines and shading repr [file supplementary_figure_3.pdf]
